# Breast Cancer Survivors' Beliefs and Preferences Regarding Technology-Supported Sedentary Behavior Reduction Interventions

**DOI:** 10.3934/publichealth.2016.3.592

**Published:** 2016-08-16

**Authors:** Gillian R. Lloyd, Sonal Oza, Sarah Kozey-Keadle, Christine A. Pellegrini, David E. Conroy, Frank J. Penedo, Bonnie J. Spring, Siobhan M. Phillips

**Affiliations:** 1 Department of Preventive Medicine, Northwestern University Feinberg School of Medicine, Chicago, IL, USA; 2 Department of Physical Medicine and Rehabilitation, Northwestern University Feinberg School of Medicine, USA; 3 Division of Cancer Epidemiology & Genetics, National Cancer Institute, Bethesda, MD, USA; 4 Department of Kinesiology, The Pennsylvania State University, University Park, PA, USA; 5 Department of Medical Social Sciences, Northwestern University Feinberg School of Medicine, Chicago, IL, USA

**Keywords:** sedentary behavior, technology, interventions, breast cancer survivors

## Abstract

**Purpose:**

Less time spent in sedentary behaviors is associated with improved health and disease outcomes in breast cancer survivors. However, little is known about survivors' interest in sedentary behavior reduction interventions and how to effectively reduce this risk behavior. The purpose of this study was to explore breast cancer survivors' interest in and preferences for technology-supported sedentary behavior reduction interventions.

**Methods:**

Breast cancer survivors (n = 279; *M_age_* = 60.7 (*SD* = 9.7)) completed a battery of online questionnaires. Descriptive statistics were calculated for all data. To examine potential relationships between demographic, disease and behavioral factors, and survivors' interest in a technology-supported sedentary behavior reduction intervention, we conducted logistic regression analyses. These same factors were examined in relation to the perceptions of the effectiveness of such intervention using multiple regression analyses.

**Results:**

On average, survivors spent 10.1 (*SD* = 4.3) hours/day in sedentary activity. They believed prolonged periods of sedentary behavior were harmful to their health (87.0%) and that reducing sedentary behavior could improve their health (88.4%). Survivors believed they should move around after 30–60 (56.7%) or ≥ 60 (29.9%) minutes of sedentary behavior and indicated they were most likely to replace sedentary behaviors with walking around (97.1%) or walking in place (73.4%). The majority of survivors (79.9%) was interested in participating in a technology-supported sedentary behavior reduction intervention and indicated they would use a smartphone application (61.3%) 2–3 times/day (48.0%), 6 to 7 days/week (52.0%). Most survivors (73.5%) believed reminders would help them decrease sedentary behavior and preferred they be delivered after sitting for 60 minutes (60.5%) via vibrations on a wrist worn activity tracker (77.3%) or text messages (54.4%).

**Conclusions:**

Technology-supported sedentary behavior reduction interventions may be feasible and acceptable to breast cancer survivors. Data regarding user preferences for content, features, delivery mode and design will aid researchers in developing sedentary interventions that are potentially more relevant and effective from the outset.

## 1. Introduction

There are approximately 3 million breast cancer survivors in the U.S. with this number expected to increase to 4 million by 2024 [1]. Breast cancer survivors have an increased risk of early mortality, comorbid conditions [2] and second primary cancers [2],[3]. Emerging evidence indicates higher levels of sedentary behavior (i.e., ≤ 1.5 metabolic equivalents (METs); i.e., sitting or reclining) [4] are associated with reduced quality of life (QOL) [5],[6], poorer physical functioning [7] and body composition [8], and increased mortality [9] among breast cancer survivors. Even among survivors who meet moderate to vigorous physical activity (MVPA; ≥ 3.0 METs; jogging, brisk walking) recommendations (i.e., 150 min/week) [10]. Despite adverse health effects associated with high levels of sedentary behavior, breast cancer survivors spend approximately 70% of their waking time sedentary [8],[10],[11] and engage in more sedentary behavior than healthy controls [10].

Too much sedentary behavior is distinct from too little MVPA as an individual can meet MVPA guidelines (i.e., 150 min/week) but still engage in a high amount of sedentary behavior [12]. Replacing sedentary behavior with MVPA may result in the greatest health benefits. However, up to 70% of breast cancer survivors do not meet MVPA recommendations [13]–[15]. As light intensity physical activity (1.6 to < 3.0 METS; i.e., activities of daily living, climbing stairs, slow walking, household chores) has been independently associated with reduced functional decline [7] and fatigue [16] and improved QOL in cancer survivors [17], substituting sedentary behavior with light intensity activities, even without increasing MVPA, may be beneficial for breast cancer survivors. Additionally, this may be a more achievable goal for survivors than meeting weekly MVPA guidelines [18]. Replacing sedentary behavior with light intensity activities may also facilitate the gradual adoption of MVPA by improving physical functioning and providing mastery experiences whereby survivors gradually sit less and move more [19]. While numerous studies have focused on increasing MVPA in breast cancer survivors, few, if any, have focused specifically on reducing sedentary behavior among breast cancer survivors [20].

Interventions focused on MVPA promotion in the absence of targeted sedentary behavior reduction do not necessarily result in changes in sedentary behavior [21]. Typical MVPA intervention approaches whereby participants are instructed to carve out a certain amount of time within their day to engage in MVPA are unlikely to change sedentary behaviors because these behaviors are more habitual and engrained in daily activities than MVPA [22]. Sedentary behavior reduction interventions will likely need to encourage breaking up sedentary behavior with short periods of light activity (e.g., standing, walking) at several time points throughout the day and, thus, may require a higher frequency of daily interventions which is less amenable to traditional on-site or telephone-based intervention modalities.

Since interventions may have to target sedentary behavior differently than MVPA, and data [23] indicates that the adoption of technology is steadily increasing, individual-level sedentary behavior reduction interventions delivered using technology (i.e., smartphones or the Internet) may be particularly useful. Further, as a result of fewer clinic visits following the completion of active treatment, survivors may feel isolated and may lack direction in how to manage their health [24]. Technology-based applications (apps) may help bridge this void by providing accessible, portable, supportive and empowering resources such as additional educational materials, target goals and useful feedback without requiring the burden of traveling to cancer care centers [25],[26]. However, before designing and testing technology-supported sedentary behavior reduction interventions, careful consideration of survivors' beliefs, interests, and preferences regarding these interventions is necessary in order to increase intervention relevance, effectiveness and uptake by survivors [27]–[29].

The purpose of the present study is to assess breast cancer survivors' interest in and preferences for technology-supported (i.e., smartphone application, website, and wearable activity tracker) sedentary behavior reduction interventions generally and with regard to specific content, features, delivery mode and design. These data will aid researchers in developing sedentary interventions that are potentially more relevant and effective from the outset.

## 2. Methods

### 2.1. Participants and Procedures

Women were recruited in July 2015 from a nationwide database of breast cancer survivors who had participated in a prior study on physical activity and QOL and agreed to be notified about future research opportunities (n = 1,366). Women were sent an e-mail describing the purpose of the present study and the eligibility criteria. Self-reported inclusionary criteria included: age ≥ 18 years, history of breast cancer, any time post-treatment completion (i.e., surgery, radiation, chemotherapy), ability to read, write and speak English, and Internet access. If interested in participating, women were instructed to click on a personalized link to a webpage containing an online screening tool. Women meeting the eligibility criteria were automatically redirected to an online informed consent, and, if they consented, they were automatically redirected to study questionnaires. Women received up to two reminders to complete the survey 7 and 14 days from the original message being sent. All study procedures were approved by the Institutional Review Board.

### 2.2. Measures

*Demographics and health history.* Participants reported their current age, marital status, race/ethnicity, income, education and height and weight to calculate body mass index (BMI). Participants rated their overall health status from poor to excellent and indicated whether they had been diagnosed with any of 18 chronic conditions (e.g. diabetes, heart disease, multiple sclerosis, depression, etc.) [30],[31]. Participants' self-reported information on their breast cancer history including the date of diagnosis, disease stage, dates and type(s) of treatment received, menopausal status at diagnosis and whether they had been diagnosed (yes/no) with a cancer recurrence.

*Technology ownership and usage:* Participants self-reported information on whether they owned a mobile phone, tablet, or computer.

*Godin Leisure Time Exercise Questionnaire* [32]. Participants indicated the frequency and average amount of time they spent engaging in strenuous (e.g., jogging), moderate (e.g., fast walking), and mild (e.g., easy walking) exercise for at least 15 minutes over the past seven days. The total weekly volume of time spent in MVPA was calculated by multiplying the weekly frequency by duration for strenuous and moderate activities. To obtain the daily average volume of MVPA, this value was divided by 7. This measure has demonstrated high reliability and validity in the general population [32] and cancer survivors [33].

*Sitting Time Questionnaire* [34]. Participants indicated the amount of time they spent sitting on a typical weekday and weekend in the following situations: traveling to and from places, while at work, while watching television, while using a computer at home, and in leisure time not including television (e.g. visiting friends, movies, dining out, etc.). Average total weekly time spent sitting was calculated by multiplying the total time spent sitting on weekdays by five and the total time spent sitting on weekends by two and summing these values. To get an average daily volume, this value was divided by 7. This measure has demonstrated good test-retest reliability and adequate validity for both total sedentary time and domain-specific sedentary time [35].

*Beliefs about sedentary behavior.* Participants answered a questionnaire created for this study to indicate their general beliefs about sedentary behavior including whether extended periods of sedentary behavior were harmful to their health and if reducing extended periods of sedentary behavior could improve their health. They were also asked to indicate their thoughts on the maximum amount of time they should spent sitting before getting up and their satisfaction with the amount of time they spend sedentary during work and leisure time. Last, participants were asked how likely they would be to replace sedentary time with specific activities (e.g. standing, calisthenics, walking in place, walking around). All items were selected for inclusion based on a thorough search of relevant literature, and responses were indicated on Likert scales, which varied depending on the specific item.

*Interest and preferences for technology-supported sedentary behavior reduction interventions.* Participants indicated their overall interest in participating in a technology-supported sedentary behavior reduction intervention and whether they believed an intervention specifically designed to help breast cancer survivors reduce sedentary behavior would be effective. They also indicated the perceived utility of a technology-supported sedentary behavior reduction intervention as well as how often and for how long each time they were willing to use a sedentary behavior reduction app. Finally, survivors reported their preferences for potential intervention features, including sedentary behavior reduction reminders and activity trackers, and intervention timing (i.e., before, during, specific post-treatment intervals). All items were selected for inclusion based on a thorough search of relevant literature and responses were indicated on Likert scales, which varied depending on the specific item.

### 2.3. Data Analysis

Descriptive statistics including means and standard deviations (i.e., continuous variables) and frequencies (i.e., categorical variables) were calculated for all data including demographic and disease characteristics, technology ownership, sedentary behavior, physical activity, technology usage, beliefs about sedentary behavior and sedentary behavior reduction intervention interests and preferences. A binary logistic regression model was conducted to examine the relationship between breast cancer survivors' interest in participating in a technology-supported sedentary behavior reduction intervention while linear regression was used to examine these same factors in relation to participants' perceptions of the effectiveness of such an intervention. For both models, the demographic covariates included age, education, annual household income, BMI, number of chronic conditions, and general health status (all continuous). The disease characteristics variables included age at diagnosis (continuous), time since treatment (continuous), disease stage (0,1,2,3,4), whether each type of treatment (chemotherapy, radiation therapy surgery) was ever received (yes/no) and whether participants had been diagnosed with a cancer recurrence (yes/no). Average total daily time spent sedentary and total weekly MVPA were also included. Each of these models also included the other outcome of interest (i.e. the interest model included perceived effectiveness and vice versa). Missing data ranged from 0% on the majority of included variables to 16.8% (household income). Preliminary analyses indicated data were missing completely at random. As such, we used mean imputation to handle missing values. All analyses were conducted in SPSS V.22 [36].

## 3. Results

### 3.1. Participant Characteristics

Of the 1,366 women approached, 279 (20.4%) responded within the first 2 weeks after which recruitment was halted due to budgetary constraints. On average, breast cancer survivors were 60.7 (*SD* = 9.7) years of age (See Table 1). The majority were White (97.1%) and non-Hispanic/Latino (98.2%). Approximately three-fourths of the sample (71.7%) had at least a college degree, and 54.4% had an annual household income ≥ $80,000. Over half (55%) of survivors were overweight or obese. The majority of women were diagnosed with early stage disease (55.2% stage 0 or I). The average time since diagnosis was 11.6 (*SD* = 5.8) years and the average time since treatment was 10.2 (*SD* = 7.3) years. A majority of women had received surgery (89.6%), radiation (72.0%), or chemotherapy (58.8%), alone, or in some combination. Further details regarding demographic and disease characteristics are presented in Table 1.

**Table 1.** Sample Demographic and Disease Characteristics (n = 279).

Factor	Frequency (%)
Marital Status	
Married/Partnered/Significant Other	78.9
Single/Divorced/Separated	15.4
Widowed	5.7
Employment Status	
Working at least Part-time	53.8
Not Working	41.3
Race	
White	97.1
Non-White	2.9
Ethnicity-Not Hispanic/Latina	98.2
Education	
< College Degree	28.3
≥ College Degree	71.7
Annual Household Income	
< $80,000	45.6
≥ $80,000	54.4
BMI *(M, SD)*	26.5 (5.7)
Normal Weight	45.0
Overweight/Obese	55.0
Overall Health Status	
Fair/Good	30.5
Very Good/Excellent	69.5
Number of Chronic Conditions *(M, SD)*	1.7 (1.5)
None	24.7
1 to 2	51.6
≥ 3	23.7
Age *(M, SD)*	60.7 (9.7)
< 65	62.7
> 65	37.3
**Breast Cancer Characteristics**	
Treatment Ever Received	
Chemotherapy	58.8
Radiation	72.0
Surgery	89.6
Other	20.4
Disease Stage	
Stage 0/I	55.2
Stage II/III	43.3
Stage IV	1.5
Time Since Diagnosis *(M, SD)*	11.6 (5.9)
< 5 Years	2.5
5 < 10 Years	50.2
> 10 Years	47.3
Diagnosed with Recurrence	14.7
Post-menopausal at Diagnosis	86.7

### 3.2. Technology Ownership and Usage

The majority (98.6%) of the sample owned a computer or smartphone (84.8%) and about two-thirds (64.5%) owned a tablet. Less than half (40.9%) of the sample owned a physical activity tracker. Of those women who owned a physical activity tracker, 28.3% reported wearing it never or less than one day, while 53.1% of owners reported wearing it at least 5 days per week. Fitbit was the most commonly owned physical activity tracker (55.6%).

### 3.3. Sedentary Behavior and Physical Activity Participation

On average, women spent 70.7 (*SD = 30.3)* total hours per week or 10.1 (*SD* = 4.3) hours/day in sedentary behavior and 187.0 (*SD* = 17.4) minutes/week or 26.7 (*SD =* 25.3) minutes per day in MVPA. About half of participants (53.4%) reported a level of physical activity that met MVPA guidelines.

### 3.4. Beliefs about Sedentary Behavior

Table 2 displays survivors' beliefs about sedentary behavior. Briefly, the majority of participants believed that extended periods of sitting or lying down was harmful to their health (87.0%) and that reducing these behaviors could improve their health (88.8%). Over half of participants (56.7%) believed they should move around after 30 to < 60 minutes of consecutive time spent sedentary and 29.9% believed they should move around after ≥ 60 minutes of sedentary behavior. Survivors who were employed at least part-time were most likely to indicate they wished they could sit or lie down less (39.2%) or did not wish to change these behaviors (21.2%) during work hours. During leisure time, 53.3% of the sample indicated they wished they could sit or lie down less, and 42.7% indicated they did not wish to change these behaviors. Survivors indicated that, if they were trying to reduce the amount of time they spend sitting, they would most likely replace sedentary behavior with walking around (97.1%) or walking in place (73.4%). Conversely, survivors were least interested in calisthenics (30.1%) in place of sedentary activities. Finally, 89.1% of the sample indicated they were unsure how likely they would be to replace sedentary behavior with standing.

**Table 2.** Breast Cancer Survivors' Beliefs About Sedentary Behavior.

Factor	Frequency (%)
Do you think sitting or lying down for extended periods of time is harmful for your health? (Yes)	87.0
Do you think reducing the amount of time you spend sitting for extended periods could help improve your health? (Yes)	88.8
What is the maximum amount of time you think you should spend sitting or lying down at home, work or in your leisure time before standing up or getting up to move around?	
< 30 minutes	13.4
30 to < 60 minutes	56.7
≥ 60 minutes	29.9
Which of the following is true regarding the amount of time you spend sitting and/or lying down at WORK?	
I wish I could be more sedentary	3.6
I wish I could be less sedentary	39.2
I do not wish to change amount of time sedentary	21.2
Not applicable	36.0
Which of the following is true regarding the amount of time you spend sitting and/or lying down during your LEISURE TIME?	
I wish I could be more sedentary	4.0
I wish I could be less sedentary	53.3
I do not wish to change amount of time sedentary	42.7
If you were trying to reduce the amount of time you spent sitting for extended periods, how likely would you be to replace sitting with each of the following activities?^a^	
Standing	
Somewhat/very likely	4.0
Very/somewhat unlikely	6.9
Not sure	89.1
Calisthenics	
Somewhat/very likely	30.1
Very/somewhat unlikely	46.9
Not sure	22.9
Walking in place	
Somewhat/very likely	73.4
Very/somewhat unlikely	14.2
Not sure	12.4
Walking around	
Somewhat/very likely	97.1
Very/somewhat unlikely	1.1
Not sure	1.8

Notes: ^a^ Responses were on 5 point Likert scale from very unlikely to very likely.

### 3.5. Interest and Preferences for Technology-supported Sedentary Behavior Reduction Interventions

Table 3 describes survivors' beliefs concerning the perceived usefulness of a technology-supported sedentary behavior reduction intervention, their willingness to participate in such an intervention and their preferences regarding potential intervention features. Overall, 79.9% of the sample indicated they would be interested in participating in a technology-supported (i.e., web, mobile phone or tablet delivered) sedentary behavior reduction intervention. About two-thirds (61.3%) of participants indicated that an app or website specifically designed to help breast cancer survivors reduce sedentary behavior would be very or somewhat effective and that they would use such an app (62.5%) or website (58.6%). Of those who indicated they would use a sedentary behavior reduction app or website, about half indicated they would use such an app or website 2–3 times per day on 6 to 7 days/week. These survivors were split almost evenly between being willing to spend 3 to 5 (33.7%) or 5 to 10+ (39.8%) minutes using the app each time. Finally, when asked to indicate how helpful they thought a technology-supported intervention would be at various time points, survivors indicated that such an intervention would be somewhat/very helpful at each of the following time points post-treatment: 6 months (77.7%), 6 months to 1 year (90.2%) and more than 1 year (89.9%). Fewer women believed an intervention would be somewhat/very helpful at the following time points: immediately post-treatment (41.0%), before treatment (50.7%) or during treatment (64.4%).

With regard to reminders to reduce sedentary behavior, about three-quarters of participants (73.5%) believed reminders to reduce sedentary behavior would help them sit less, and most (60.5%) preferred reminders after sitting for 60 minutes. The most favored delivery modes for these reminders were vibrations on a wrist-worn activity tracker (77.3%) and text message (54.5%). Additionally, survivors wanted some ability to control reminders as 93.1% indicated they wanted to be able to turn “off” reminders for a specified period of time (e.g., during a meeting), 68.5% wanted the ability to “snooze”/delay reminders, and 58.8% wanted to be able to ignore reminders. Finally, 64.5% of the sample believed a physical activity tracker would help decrease the amount of time breast cancer survivors spend sitting. In terms of activity tracker feedback, 67.4% wanted feedback on time spent sitting and 48.7% wanted feedback on time spent standing.

**Table 3.** Breast Cancer Survivors' Interest and Preferences for Technology-Supported Sedentary Behavior Reduction Interventions (n = 279).

Factor	Frequency (%)
Would you be interested in participating in an intervention to reduce sitting time that was delivered via mobile phone, tablet or website, and did not require you to have any on-site visits to a cancer center, university or recreation center? (Yes)	79.9
**Usefulness of Technology-Supported Intervention**	
How effective do you think an app or website specifically designed to help breast cancer survivors reduce their sitting would be for reducing time spent sitting? ^a^	
Very effective/Somewhat effective	61.3
Not effective/Not effective at all	6.4
Not sure	32.3
If there were a MOBILE APP available specifically to help breast cancer survivors increase their exercise available for free, would you download it?	
Yes	62.5
No	17.0
Don't know	20.6
If there were a WEBSITE available specifically to help breast cancer survivors increase their exercise available for free, would you use it?	
Yes	58.6
No	15.1
Don't know	26.3
**Frequency and Duration of Sedentary Behavior Reduction App**	
If you were to be given an app or website specifically designed to help you reduce the amount of time you spend sitting how many days per week, in an average week, do you think you would open up the app or website?	
< 3 days	23.6
4–5 days	24.3
6–7 days	52.0
How many times would you be willing to interact (e.g. enter data, look at feedback, post messages, etc.) with the app or website on these days?	
1 time/day	24.8
2–3 times/day	48.0
4–5+ times/day	27.2
How much time would you be willing to interact with the app or website each time (e.g. enter data, look at feedback, post messages, etc.)?	
< 1–3 min	26.5
3–5 min	33.7
5–10+ min	39.8
**Preferences for Timing of Intervention**	
If there was an intervention to reduce sitting time for women diagnosed with breast cancer, how helpful do you think it would be at each of these time points? ^b^	
Before treatment	
Somewhat helpful/helpful	50.7
Not very helpful/not helpful	22.3
Not sure	27.0
During treatment	
Somewhat helpful/helpful	64.4
Not very helpful/not helpful	13.7
Not sure	21.9
Immediately post treatment	
Somewhat helpful/helpful	41.0
Not very helpful/not helpful	6.9
Not sure	15.5
6-months post-treatment	
Somewhat helpful/helpful	77.7
Not very helpful/not helpful	1.1
Not sure	7.9
6-months to 1 year post-treatment	
Somewhat helpful/helpful	90.2
Not very helpful/not helpful	1.5
Not sure	8.3
More than 1 year post-treatment	
Somewhat helpful/helpful	89.9
Not very helpful/not helpful	2.9
Not sure	12.3
**Preferences for Sedentary Behavior Reduction Reminders**	
Do you think reminders to stand or move around when you had been sitting for extended periods of time would help you sit less? (Yes)	73.5
How many reminders would you be willing to receive each day?	
1–3	55.4
4–6	27.7
7 and above	16.9
How often do you think reminders should be delivered?	
When sedentary for 90+ minutes	20.2
When sedentary for 60 minutes	60.5
When sedentary for 30 minutes	19.5
How would you like these reminders delivered? (Mark all that apply)	
Vibrations on a wrist-worn activity tracker	77.3
Text-message	54.5
Pop-up reminder on computer	34.8
Automated pop-up telephone message	10.7
Changes in cell phone screen brightness or performance	6.9
Would you like to be able to…	
Turn “off” for a specified period of time? (Yes)	93.1
“Snooze” or delay reminders? (Yes)	68.5
Ignore reminders? (Yes)	58.8
**Preferences for Activity Trackers**	
How much do you think an activity tracker such as a pedometer (step counter), FitBit, Jawbone, or smart watch would help breast cancer survivors REDUCE THE AMOUNT OF TIME THEY SPEND SITTING? ^c^	
A lot/Somewhat	64.5
Not much at all/Not much	3.6
Not sure	31.9
If you were to use an activity tracker that could be specifically designed to fit your needs, what type of information would you like to receive feedback on? (Mark all that apply.)	
Time spent sitting	67.4
Time spent standing	48.7

Notes: ^a^ Responses were on 5 point Likert scale from very effective to not effective at all; ^b^ Responses were on 5 point Likert scale from very helpful to not helpful; ^c^ Responses were on 5 point Likert scale from a lot to not much at all.

### 3.6. Factors Associated with Interest and Perceived Effectiveness of Technology-supported Sedentary Behavior Reduction Interventions

Interest in a technology-supported sedentary behavior reduction interventions (χ^2^ = 37.4, *df* = 15, *p* = 0.001) and beliefs about effectiveness (F(15,263) = 5.15, *p* < 0.001, R^2^ = 0.18), were not independently associated with any of the demographic or diseases characteristics explored, daily average sitting time, or weekly physical activity participation. However, individuals who believed a sedentary behavior reduction app would be effective were significantly (OR = 5.1; 95% CI = 2.4–11.3; *p* < 0.001) more likely to indicate that they were interested in participating in such an intervention. Finally, individuals who were interested in participating in a sedentary behavior reduction intervention were also more likely *(β* = 0.76; *p* < 0.001) to believe it would be effective (data not shown).

## 4. Discussion

The purpose of the present study was to examine breast cancer survivors' beliefs about sedentary behavior as well as their interest in, and preferences for, technology-supported sedentary behavior reduction interventions. Overall, the majority of participants believed engaging in sedentary behavior for extended periods is harmful to their health (87.0%) and reducing time spent sitting or lying down could improve their health (88.8%). The majority of participants (79.9%) also indicated they would be interested in participating in a technology-supported sedentary behavior intervention. About two-thirds of participants agreed that an app or website specifically designed to help breast cancer survivors reduce sedentary behavior would be effective and that they would use such an app or website. These findings indicate that technology-supported sedentary behavior reduction interventions are appealing to breast cancer survivors. Further, these data provide insight into survivors' preferences for specific program features, which should be taken into consideration when designing sedentary behavior reduction interventions for this group.

While the majority of survivors believed that extended periods of sedentary behavior are harmful to their health, a much lower proportion wished they could sit less either at work or during their leisure time. Interestingly, almost half did not wish to change their sedentary behavior during their leisure time, and ∼20% did not wish to reduce their sedentary behavior during work. These incongruous findings may indicate survivors did not wish to change their behavior because they already engaged in low levels of sedentary behavior. However, our data suggest otherwise as survivors reported spending an average of 10.1 hours/day sedentary. Therefore, survivors may simply be unaware of how much time they spend sedentary [21],[37], or there may be other factors (e.g. symptoms, motivation, beliefs about what types of activities they can replace sedentary behavior with) influencing their desire to change their sedentary behaviors. Future research should seek to understand this disconnect between sedentary beliefs and behaviors as it may have implications for intervention success.

Existing evidence suggest that breaking up prolonged bouts of sedentary behavior is beneficial for a variety of health outcomes in the general population [38]–[41]. Although there is no consensus on how frequently breaks in sedentary behavior should occur, it appears that more frequent breaks may be more beneficial [42]. Our study adopted a patient-centered approach to try to understand what types of sedentary behavior reduction strategies (i.e. replacing sitting with standing, promoting short bouts of walking, etc.) may be most salient to breast cancer survivors. More than half of participants (56.7%) believed they should move around after 30 to < 60 minutes of sedentary behavior. The majority of survivors indicated they were somewhat/very likely to replace sedentary behavior with walking around or walking in place, and they were least interested in calisthenics. While many existing sedentary behavior reduction interventions aim to reduce sedentary time by replacing sitting with standing [43]–[45], 89.1% of our sample reported feeling “unsure” about replacing sedentary behavior with standing. There may be several reasons for this finding. For example, survivors may often be in situations where they feel social norms discourage replacing sitting with standing (e.g., in a movie theater, dining,) or where logistical constraints impede standing (e.g., desk jobs, driving,) [46]. Further, survivors may believe that standing provides insufficient health benefits to be worthwhile, and thus, further education may be required to motivate behavior change [47]. This finding warrants additional exploration to better understand the context of breast cancer survivors' time spent sedentary and factors (e.g.,. knowledge, beliefs, motivation) that may be related to their uncertainty regarding standing as a replacement behavior. Additionally, these data indicate it is important to develop a better understanding of the most preferred activities to replace sedentary behavior and effective strategies for integrating these behaviors into different contexts prior to designing an intervention.

The majority of the sample, regardless of demographic or disease characteristics, physical activity, or time spent sedentary expressed an interest in participating in a technology-supported sedentary behavior reduction intervention that used an app or website which is consistent with studies in other survivor groups [48]. These data suggest that even older adults, who make-up the majority of the breast cancer survivor population [49], may be interested and willing to use technology-supported interventions. The majority of survivors indicated they were willing to use the app 2–3 times per day for at least 3 to 5 minutes at least 4–5 days per week. While this is promising, survivors were more skeptical about whether such an app or website would be effective for reducing sedentary behaviors or whether they would actually use it. This implies that survivors may be willing to try a sedentary behavior reduction app or website, even if they are unsure of its effectiveness, but that interventions using such tools should be carefully designed to maximize adherence and ensure adequate engagement. Further, these findings indicate intervention content needs to be relatively brief. Finally, most survivors believed that the optimal timing to offer a technology-supported sedentary behavior reduction intervention would be at least six-months post-treatment. Interestingly, about two-thirds also believed that such an intervention would be helpful during treatment. As survivors may experience unique barriers (e.g., increased symptom burden) and benefits (e.g., maintaining physical function, improving cardiometabolic biomarkers) to reducing sedentary behavior depending on their time since diagnosis, future research should explore optimal intervention timing in terms of both feasibility/acceptability and maximizing health outcomes.

Our findings indicate that about three-quarters of breast cancer survivors believed reminders or prompts to reduce sedentary behavior would help them sit less which is consistent with another study examining sedentary behavior intervention preferences in prostate cancer survivors [48]. While most survivors believed they should break up their sedentary behavior after 30 to < 60 minutes of consecutive sitting, the majority preferred to have reminders delivered after ≥ 60 minutes of sedentary behavior. While prior research indicates it may be ideal to break up sedentary behavior more frequently [50],[51], it is important to consider participant burden when designing these interventions and to weigh the potential cons (e.g., participant burden, de-sensitization) of more frequent prompts versus the potential health benefits of prompts delivered every 60 minutes [52]. The most popular reminder delivery modes were vibrations on a wrist-worn activity tracker and text messages, which suggests that survivors want reminders that are private and not very invasive or disruptive. Finally, most survivors wanted to be able to have some control over reminders (i.e. snoozing, turning “off” for specified periods, ignoring). Future research should seek to understand the context and frequency of situations in which survivors would want to control reminders and how this may influence intervention outcomes including sedentary behavior reduction and potential health benefits. Machine-learning algorithms [53] may be particularly useful in this context as they could “learn” survivors' preferences based on the timing and frequency of an individual's pausing, delaying or turning off reminders and send prompts that are delivered at the most appropriate times for a given individual on a given day [54]. These algorithms could also be useful for providing specific suggestions for activities to replace sedentary behavior. For example, if a survivor always indicated they replaced sedentary behavior with small walks, the system could “learn” to send tailored strategies and prompts focused on replacing sedentary behavior with these short walks [55]. This, combined with tailored timing of reminders, could, in turn, increase intervention uptake, adherence and effectiveness.

Self-monitoring sedentary behavior as an intervention strategy poses many challenges. Sedentary behavior may be more “automatic” and engrained in everyday life than physical activity [9],[45]. Thus, many individuals are unaware of how much time they spend sitting, which has been supported by studies demonstrating that individuals tend to underreport sedentary time compared to objective measures [56]–[60]. About two-thirds of the breast cancer survivors in our sample thought self-monitoring via feedback from a physical activity tracker would help them decrease sedentary behavior. However, while commercially available activity monitors have demonstrated adequate validity for physical activity assessment [61],[62], they have not been well-validated to assess or monitor sedentary behavior. Limitations of using commercially available activity trackers for sedentary behavior assessment and monitoring include: inability to distinguish between non-wear and sitting as well as postural changes (i.e. sitting v. standing) due to their wrist placement [8],[21],[37],[44],[51],[63]–[66]. Therefore, future research is warranted to understand and test whether these devices could be used to accurately assess time spent sitting and, in turn, deliver reminders to prompt survivors to sit less and move more throughout the day.

Results of this study should be interpreted in the context of its limitations. First, our sample consisted of mostly White, high income, long-term breast cancer survivors. Additionally, sample recruitment and data collection were conducted using the Internet. Thus, our findings may not be representative of individuals who are less technologically savvy whose beliefs, preferences and needs may differ from the current sample. Thus, examining beliefs about sedentary behavior and interest in and preferences for technology-supported sedentary behavior reduction interventions in more diverse breast cancer survivor groups (i.e. African American, Hispanic, younger) who are at various stages since diagnosis and vary in their usage and experience with technology is warranted. Second, our study primarily focused on individual-level, general sedentary behavior change strategies. While there is limited evidence on which factors predict sedentary behavior among cancer survivors [67],[68], it is likely that individual (i.e. goal-setting; self-efficacy; symptoms) [22],[37],[69], environmental/structural (i.e. home, workplace, community) and social (i.e. social norms) factors [68] all influence these behaviors. Exploring these multi-level influences as well as preferences for interventions that target these factors in different contexts (e.g. home, work, leisure time, etc.) are important next steps in understanding what intervention components may be feasible, acceptable and effective for breast cancer survivors in what contexts. To this end, it is also important to understand which behavioral theories (i.e. social cognitive theory, dual process theory) may be most appropriate for understanding sedentary behavior within different contexts to further guide intervention development. Research utilizing focus groups or intensive longitudinal data collection in real-time (i.e. ecological momentary assessment) could provide insight into the context of survivors' sedentary behavior, the potential antecedents of sedentary behavior, and how these factors may change throughout the cancer experience. Finally, this study is a cross-sectional observational study that used self-reported assessments of sedentary behavior. Future research should test potential intervention components using A-B quasi experimental [63], multiphase optimization strategy (MOST) [70], and sequential multiple assignment (SMART) designs [70],[71]. These trial designs are rigorous and allow for rapid studies to identify and adapt the most effective sedentary behavior reduction intervention components, or sequence of components, to answer the questions of what works for whom, in what contexts, and for what outcomes [72].

Despite these limitations, our study also has several strengths. To the best of our knowledge, the present study is the first study to examine breast cancer survivors' beliefs about sedentary behaviors as well as their interest in and preferences for technology-supported sedentary behavior reduction interventions. Focusing specifically on sedentary behavior reduction, and not physical activity promotion, is necessary because sedentary behaviors are distinct from too little exercise [73]. Interventions designed to target one behavior may not necessarily result in significant changes in the other [21]. Additionally, engaging stakeholders prior to designing a sedentary behavior reduction intervention may help increase its relevance and effectiveness [72],[74]. Finally, the study sample was a relatively large, nationwide sample that included a wide range of disease and treatment characteristics indicating findings may be relevant to many breast cancer survivors.

In conclusion, breast cancer survivors reported high levels of sedentary behavior, recognized the potential adverse effects of sedentary behavior, and many expressed an interest in participating in a technology-supported sedentary behavior reduction intervention. These findings indicate that a technology-supported sedentary behavior reduction interventions may be feasible and acceptable to breast cancer survivors. These findings also highlight: a) the importance of engaging survivors in designing these interventions and b) the need for future research in this area to further understand what specific interventions components may be most feasible, acceptable and effective for reducing sedentary behavior and improving associated health outcomes.
